# Histone methyltransferase KMT2D targets the SPOP-G3BP1 axis to enhance AR stability and drive castration-resistant prostate cancer progression

**DOI:** 10.1186/s43556-025-00354-8

**Published:** 2025-11-17

**Authors:** Haoran Wen, Maierhaba Maheremu, Kaidi Zhang, Liuru Bao, Mayao Luo, Yifan Zhang, Yuanpeng Liao, Manli Zhou, Chenwei Wu, Shidong Lv, Xiaofu Qiu, Qiang Wei

**Affiliations:** 1 Department of Urology, Nanfang Hospital, Southern Medical University, Guangzhou, Guangdong 510515 China; 2 The Affiliated Guangdong Second Provincial General Hospital of Jinan University, Guangzhou, 510317 China; 3 Department of Urology, Ganzhou Hospital-Nanfang Hospital, Southern Medical University, Ganzhou, 341000 China; 4 Department of Urology, Guangdong Provincial People’s Hospital, Guangdong Academy of Medical Sciences, Guangdong Cardiovascular Institute, Southern Medical University, Guangzhou, Guangdong 510080 China

**Keywords:** Androgen receptor, Castration-resistant, Methylation, Signaling, Ubiquitination

## Abstract

Castration-resistant prostate cancer (CRPC) poses a significant clinical challenge, characterized by limited therapeutic options and unfavorable prognosis, particularly among elderly men. Reactivation of androgen receptor (AR) signaling remains the principal driver of CRPC cell survival and tumor progression even under castrated levels of serum androgen. Lysine methyltransferase 2D (KMT2D) has been established as a key oncogenic driver in prostate cancer, promoting tumor progression via multiple pathways. However, its functional interaction with the AR signaling axis in the context of CRPC remains incompletely understood. In this study, we demonstrate that KMT2D substantially upregulates AR protein levels, thereby reactivating AR signaling under castration conditions. Mechanistically, KMT2D employs its histone methyltransferase function to transcriptionally enhance the expression of G3BP stress granule assembly factor 1 (G3BP1). Upregulated G3BP1 subsequently suppresses the activity of the E3 ubiquitin ligase Speckle Type BTB/POZ protein (SPOP), leading to diminished AR ubiquitination and impaired proteasomal degradation. Furthermore, we explored a novel combination therapy involving the histone methyltransferase inhibitor MI-503 and enzalutamide in AR-positive and AR splice variant-positive cell lines. Our results confirmed the synergistic therapeutic effects of this combination, which can continue to inhibit the AR signaling pathway during the CRPC stage, thereby delaying disease progression. Taken together, our findings elucidate a critical KMT2D/G3BP1/SPOP/AR regulatory axis in prostate cancer progression and propose that targeted inhibition of histone methylation in combination with anti-androgen therapy represents a promising strategy for the management of advanced prostate cancer.

**Supplementary Information:**

The online version contains supplementary material available at 10.1186/s43556-025-00354-8.

## Introduction

As one of the most frequently diagnosed cancers in men worldwide, prostate cancer (PCa) is highly dependent on androgen receptor (AR) signaling for its development [1]. Consequently, androgen deprivation therapy (ADT) is widely adopted as the first-line option for metastatic PCa [2]. However, PCa relapses within 2–3 years and progresses to a fatal stage termed castration-resistant prostate cancer (CRPC). This stage is characterized by resistance to ADT due to AR reactivation and results in a poor prognosis [3–7]. During the CRPC stage, AR exhibits alterations in its structure, expression, and subcellular localization [8]. Multiple mechanisms, including *AR* mutations, splice variants, overexpression, and nuclear enrichment, mediate the reactivation of AR at castrate levels. Therefore, devising strategies to retarget the reactivated AR is key to developing CRPC therapeutics [6, 9–12].

Lysine methyltransferase 2D (KMT2D) is a histone methyltransferase that activates enhancers by mediating monomethylation at histone H3 lysine 4 (H3K4me1), thereby regulating the expression of downstream genes. KMT2D has been implicated in the occurrence and development of several types of malignancies [13, 14]. In previous studies, we identified *KMT2D* as a key pathogenic gene in PCa. It is not only highly mutated and overexpressed in tumor patients but also promotes the proliferation and metastasis of PCa through various mechanisms, including activation of the leukemia inhibitory factor receptor (LIFR), Kruppel-like factor-4 (KLF4) and antioxidant pathways [15–17]. However, in previous studies, we used AR-negative cell lines and overlooked the potential association between KMT2D and AR, which is the most important mechanism driving the PCa. Therefore, whether KMT2D is involved in AR regulation and mediates CRPC progression remains unclear.

The speckle type BTB/POZ protein gene (*SPOP*) is a frequently mutated gene in PCa that serves as a crucial tumor suppressor by functioning as a ubiquitin ligase, mediating the intracellular degradation of AR [18]. Recently, G3BP stress granule assembly factor 1 (G3BP1) was reported to inhibit SPOP by competing with its substrates for binding to the MATH domain, thereby preventing SPOP-mediated ubiquitination and degradation [19]. Here, we show, for the first time, that KMT2D is involved in the reactivation of AR in CRPC. Through the SPOP–G3BP1 axis, KMT2D epigenetically activates G3BP1, which subsequently prevents AR ubiquitination and degradation, leading to its overexpression in CRPC. Moreover, we demonstrate that a combination of the histone methyltransferase inhibitor MI-503 and the second-generation AR antagonist enzalutamide synergistically inhibits the AR signaling pathway by targeting both AR expression and activity. This combination effectively suppresses the progression of CRPC, offering a novel therapeutic strategy for advanced prostate cancer.

## Results

### KMT2D regulates AR stability through ubiquitination in PCa

**Fig. 1:**
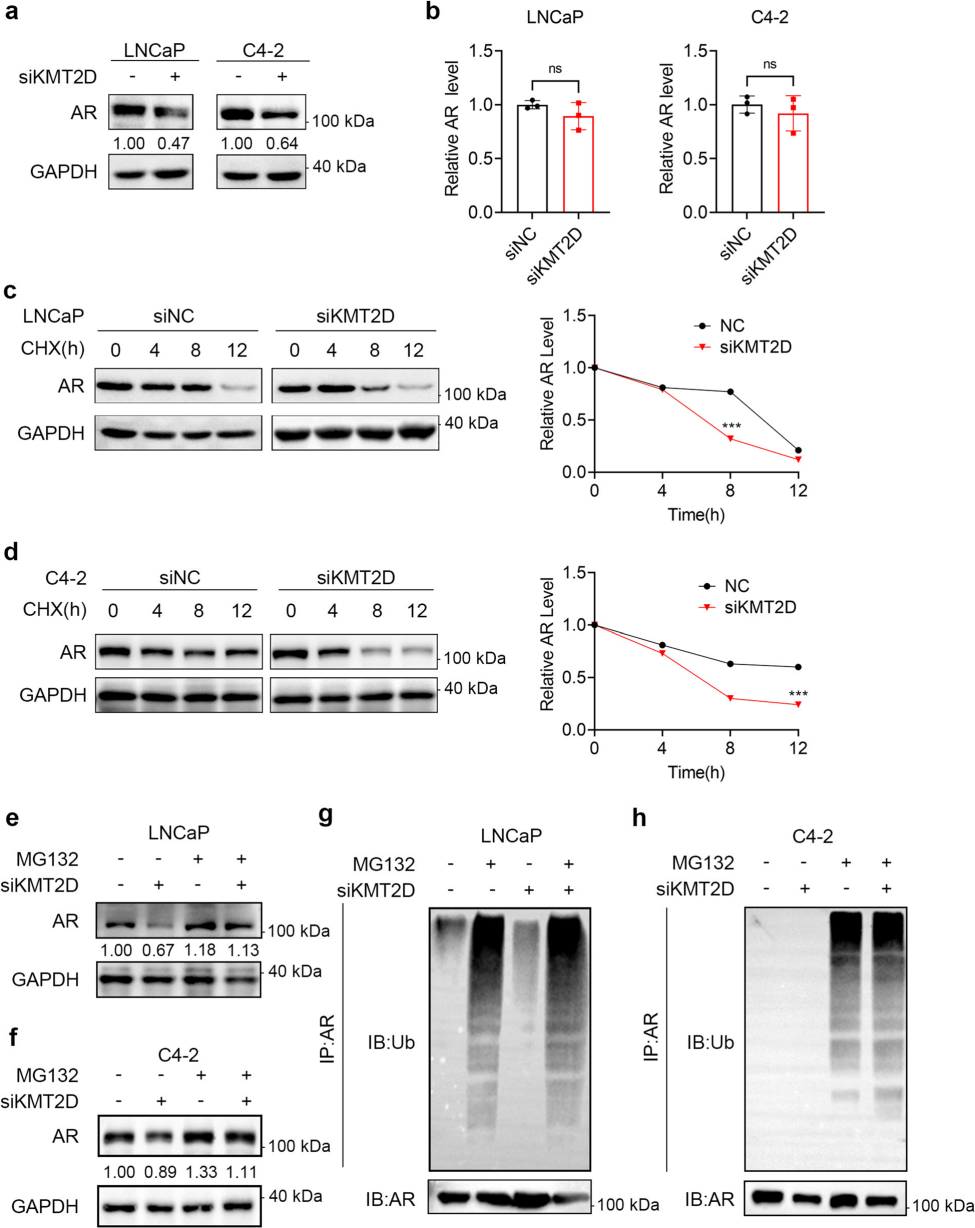
KMT2D regulates androgen receptor (AR) stability through ubiquitination in prostate cancer. **a**, **b** siNC or siKMT2D was transfected into LNCaP (**a**) or C4-2 (**b**) cells. After 48 h, total RNA was collected from the cells, and RT-qPCR was performed to detect changes in *AR* mRNA levels. After 72 h, total protein was collected from the cells, and western blotting was performed to detect AR protein levels. **c**, **d** LNCaP (**C**) and C4-2 (**d**) cells were transfected with siNC or siKMT2D and 48 h later, the cells were treated with cycloheximide (CHX). After incubation for 0, 4, 8, and 12 h, total cellular protein was extracted for protein immunoblot analysis to detect the proteins shown in the figure. All the data were obtained from three independent replicates. **e**, **f** siNCs or siKMT2D were transfected into LNCaP (**e**) or C4-2 (**f**) cells. After 48 h, the cells were treated with either dimethyl sulfoxide (DMSO) or 10 μM of MG132 for an additional 24 h before extraction of total cellular protein. AR levels were assessed via western blotting. **g**, **h** LNCaP (**g**) and C4-2 (**h**) cells were transfected with plasmids expressing hemagglutinin-tagged ubiquitin (HA-Ub) and Flag-tagged AR (Flag-AR), along with siRNA targeting either siNC (non-targeting control) or siKMT2D. At 48 h post-transfection, the cells were treated with the proteasome inhibitor, MG132, for an additional 24 h before extraction of total cellular protein. A co-immunoprecipitation (co-IP) assay was performed using an antibody specific for AR, followed by western blotting to detect the ubiquitination status of AR. All the data were obtained from three independent replicates. *P < 0.05; **P < 0.01; ***P < 0.001; ns not significant. All experiments were conducted in triplicates, and representative data are presented

To investigate the clinical relevance of KMT2D expression in PCa progression, we analyzed two publicly available gene expression datasets. In the GSE35988 dataset, *KMT2D* mRNA levels were significantly elevated in CRPC samples compared with those in localized PCa samples (Fig. S1a). Consistently, analysis of the GSE70770 dataset revealed markedly higher *KMT2D* expression in CRPC than in localized PCa (Fig. S1b). These findings indicate that *KMT2D* is upregulated during the progression from localized PCa to CRPC. To clarify the role of KMT2D in AR-positive PCa, we knocked it down in LNCaP and C4-2 cell lines using siRNA and evaluated the AR expression levels using western blotting. These cells harbor wild-type (WT) *SPOP* gene [20]. Although LNCaP is a hormone-sensitive PCa cell line and C4-2 is a castration-resistant PCa cell line, we found that after KMT2D knockdown, AR protein levels in LNCaP and C4-2, two PCa cell lines at different stages that are dependent on the AR pathway for proliferation, were significantly reduced, being approximately 0.47- and 0.64-fold of the control levels, respectively (Fig. 1a). We used Reverse Transcription quantitative Polymerase Chain Reaction (RT-qPCR) to determine the changes in *AR* mRNA levels after knocking down KMT2D in LNCaP and C4-2 cells. No significant changes in *AR* mRNA levels compared with those in the control LNCaP or C4-2 groups were observed, which indicated that KMT2D did not affect *AR* transcription (Fig. 1b). To further investigate the regulatory role of KMT2D in AR signaling, we knocked down KMT2D in 22Rv1 cells and examined the expression of full-length AR and its splice variant AR-V7. Western blot analysis revealed that KMT2D depletion markedly reduced the protein levels of both AR and AR-V7 (Fig. S1d). In addition, the levels of H3K4me1, a histone mark associated with the methyltransferase activity of KMT2D, were decreased, indicating effective knockdown and functional impairment of KMT2D. The regulation of AR protein levels by KMT2D may be at the translation or post-translational level. We knocked down KMT2D in LNCaP and C4-2 cells and measured the half-life of the AR to determine the effect of KMT2D on AR degradation. Following siRNA-mediated KMT2D knockdown, AR degradation occurred much faster, and its half-life was markedly reduced compared with the control group (Fig. 1c, d). These results indicated that KMT2D affects the half-life of AR and regulates its protein levels by inhibiting AR degradation.

In PCa cells, AR is mainly degraded via the ubiquitin–proteasome pathway. We speculated that the accelerated degradation of AR after KMT2D knockdown might be associated with its increased ubiquitination. To confirm this notion, we used MG132, a proteasome inhibitor, for recovery experiments. The AR protein levels decreased after KMT2D knockdown, and this change was restored by treatment with proteasome inhibitor (Fig. 1e, f). Therefore, we believe that KMT2D inhibits the ubiquitination and degradation of AR. We performed co-immunoprecipitation experiments to detect the ubiquitination levels of AR in PCa. MG132 treatment resulted in the inhibition of AR degradation via the ubiquitin–proteasome pathway and increased the AR ubiquitination. However, after KMT2D knockdown alone, the AR ubiquitination levels increased compared with those in the control group, and after MG132 treatment, the ubiquitination levels increased further (Fig. 1g, h).

### KMT2D indirectly regulates E3 ubiquitin ligase SPOP to affect AR stability

**Fig. 2:**
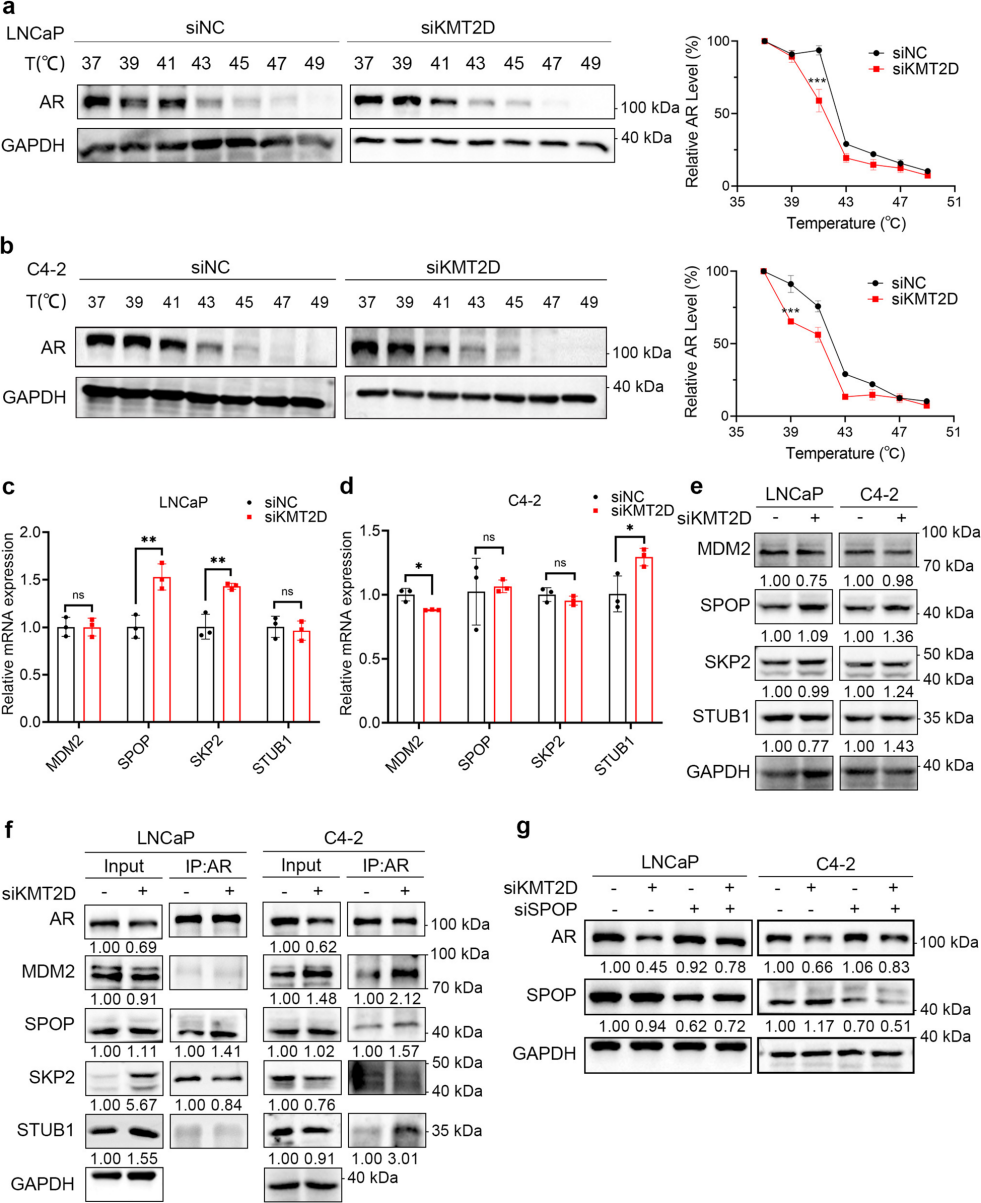
KMT2D indirectly regulates E3 ubiquitin ligase SPOP to affect androgen receptor (AR) stability. **a**, **b** Cell-based thermal shift assay (CETSA) was used to analyze the effect of enzalutamide on AR stability in C4-2 (**a**) and LNCaP (**b**) cells. The cells were transfected with either siNC (non-targeting siRNA control) or siKMT2D. **c**, **d** KMT2D was silenced in LNCaP (**c**) and C4-2 (**d**) cell lines and the mRNA levels of known AR-associated E3 ubiquitin ligases were determined via qPCR. *GAPDH* was used as the internal reference gene. **e** Protein levels of AR-associated E3 ubiquitin ligases after KMT2D silencing in LNCaP and C4-2 cell lines. **f** Western blot analysis for AR and E3 ubiquitin ligases after KMT2D silencing in LNCaP and C4-2 cells. **g** AR and SPOP were assessed via western blotting after silencing KMT2D and/or SPOP in LNCaP and C4-2 cells. All experiments were conducted in triplicates, and representative data are presented. *P < 0.05; **P < 0.01; ***P < 0.001; ns not significant. All experiments were conducted in triplicates, and representative data are presented

In prostate cancer cells, AR is produced in the cytoplasm and translocated to the nucleus upon androgen binding. It binds to DNA with the help of cofactors, and acts as a transcription factor, promoting the expression of downstream genes. The cellular thermal shift assay (CETSA) detects changes in the interactions of proteins in living cells based on changes in their thermal stability. The knockdown of KMT2D in LNCaP and C4-2 cells shifted the melting curve for AR and altered its thermal stability (Fig. 2a, b). The CETSA results indicated that KMT2D affected the interaction of the AR with other proteins. Therefore, KMT2D likely affects the binding of AR to the E3 ubiquitin ligase, which mediates AR ubiquitination, thereby regulating the AR protein levels.

We silenced KMT2D in LNCaP and C4-2 cells and examined the mRNA levels of the AR-related E3 ubiquitin ligases *SPOP*, Mouse double minute 2 homolog(*MDM2)*, S-phase kinase-associated protein 2(*SKP2)*, and STIP1 homology and U-box containing protein 1(*STUB1)* via quantitative Polymerase Chain Reaction (qPCR). After KMT2D knockdown, the expression of *SPOP* and *SKP2* increased in LNCaP cells whereas the expression of *STUB1* increased and that of *MDM2* slightly decreased in C4-2 cells. However, none of these changes exceeded a twofold difference. We consider such modest changes at the transcriptional level to be of limited biological significance (Fig. 2c, d). Furthermore, western blot analysis did not reveal any significant differences in the protein levels of these E3 ubiquitin ligases, indicating that KMT2D does not directly regulate the transcription or protein expression of AR-related E3 ubiquitin ligases (Fig. 2e, S2a).

Based on these findings, we propose the following hypothesis: KMT2D indirectly affects AR-related E3 ubiquitin ligase and further affects AR ubiquitination and its subsequent degradation. After KMT2D knockdown, only the degree of binding of SPOP to the AR was significantly enhanced by 1.41-fold in LNCaP cells and 1.57-fold in C4-2 cells, which indicates that KMT2D enhances AR ubiquitination by affecting the SPOP activity (Fig. 2f). Silencing of KMT2D alone led to a significant decrease in AR protein levels, which was consistent with our previous observations. However, when SPOP was silenced after KMT2D knockdown, the AR protein levels recovered significantly (Fig. 2g, S2b-c). These results strongly indicate that SPOP is a key E3 ubiquitin ligase downstream of KMT2D involved in regulating AR ubiquitination.

### KMT2D regulates G3BP1 to inhibit the SPOP activity

**Fig. 3:**
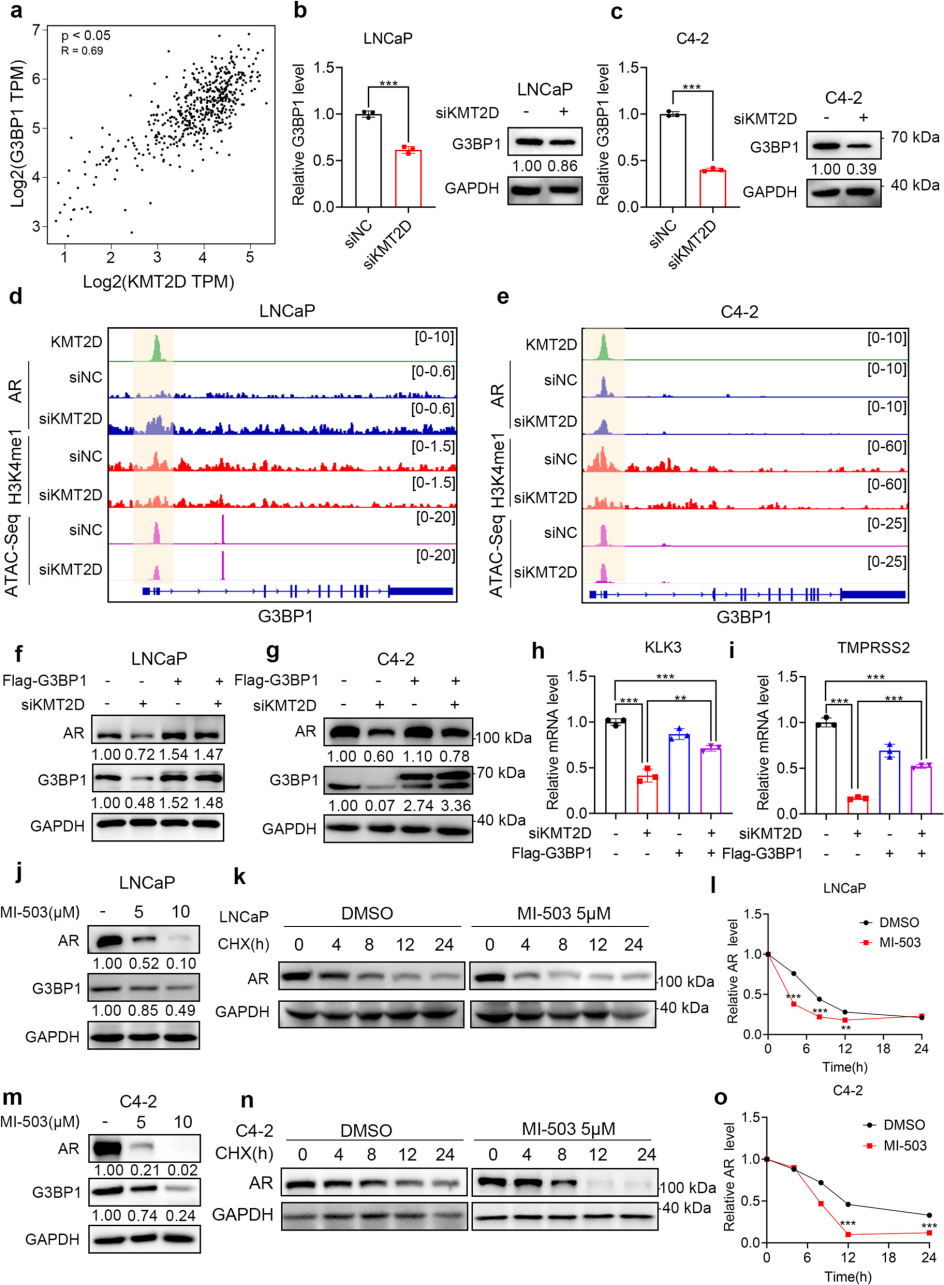
KMT2D regulates G3BP1 to inhibit the SPOP activity. **a** Correlation analysis of *KMT2D* and *G3BP1* mRNA expression in prostate adenocarcinoma samples from the Cancer Genome Atlas (TCGA) using cBioPortal. TPM values were log2-transformed. Pearson’s correlation coefficient (*R*) and *P*-value are shown. **b**, **c** The mRNA and protein levels of G3BP1 after KMT2D silencing in LNCaP (**b**) and C4-2 (**c**) cells. *GAPDH* was used as the internal reference gene. **d**, **e** H3K4me1 binding peaks in the region upstream of G3BP1 in LNCaP (**d**) and C4-2 (**e**) cells. **f**, **g** AR expression in LNCaP (**f**) and C4-2 (**g**) cells after KMT2D silencing and/or G3BP1 overexpression. **h**, **i** Activity of the AR axis after KMT2D silencing and G3BP1 overexpression in LNCaP (**H**) and C4-2 (**i**) cells. **j**, **m** LNCaP (**j**) and C4-2 (**m**) cells were treated with different concentrations of MI-503, and AR or G3BP1 expression was detected via western blotting. **k**, **l**, **n**, and **o** LNCaP (**k**, **l**) and C4-2 (**n**, **o**) cells were treated with MI-503; 48 h later, the cells were treated with CHX. After incubation for 0, 4, 8, and 12 h, total cell protein was extracted for western blotting analysis to detect the proteins shown in the figures. *P < 0.05; **P < 0.01; ***P < 0.001. All experiments were conducted in triplicates, and representative data are presented

To further investigate whether KMT2D regulates AR protein levels via G3BP1 and the underlying mechanism, we first analyzed transcriptomic data from TCGA-PRAD and observed a positive correlation between KMT2D and G3BP1 expression (Fig. 3a). Consistently, KMT2D knockdown markedly decreased G3BP1 protein levels in LNCaP and C4-2 cells (0.86-fold and 0.39-fold of control, respectively; Fig. 3b–c). Assay for transposase-accessible chromatin using sequencing (ATAC-seq) and cleavage under targets and tagmentation (CUT&Tag) tracks at the G3BP1 locus demonstrated that KMT2D depletion decreased chromatin accessibility and H3K4me1 enrichment at regulatory regions (Fig. 3d–e). Notably, AR occupancy at these regions did not show appreciable changes upon KMT2D knockdown, suggesting that KMT2D primarily affects G3BP1 transcription through modulation of chromatin state and histone modification (Fig. 3d–e).

Functionally, Ectopic expression of Flag-tagged G3BP1 led to an increase in AR protein levels—1.54‑fold of control in LNCaP and 1.10-fold in C4-2 cells. Furthermore, in KMT2D-knockdown cells, overexpression of G3BP1 partially restored AR protein levels relative to those in cells with KMT2D knockdown alone (Fig. 3f–g, S2f-g). To investigate the roles of KMT2D and G3BP1 in AR signaling, we overexpressed G3BP1 following KMT2D knockdown. As shown in Fig. 3h, i, KMT2D silencing significantly reduced the mRNA levels of AR downstream genes Kallikrein-related peptidase 3(*KLK3)* and Transmembrane protease, serine 2(*TMPRSS2)*. Overexpression of G3BP1 following KMT2D knockdown partially rescued the reductions in *KLK3* and *TMPRSS2* expression. These results indicate that G3BP1 overexpression reverses the decrease in the expression of AR downstream genes caused by KMT2D knockdown to a certain extent and partially restores the activity of the AR signaling pathway, which may be achieved through the inhibitory effect of G3BP1 on the SPOP activity.

MI-503 is an inhibitor of methylation. Given that KMT2D is a histone methyltransferase that plays a key role in regulating chromatin status and gene expression, we speculated that MI-503 could inhibit KMT2D and play a potential therapeutic role in PCa. As the concentration of MI-503 increased, AR protein levels showed a downward trend and G3BP1 protein levels showed a similar pattern (Fig. 3j, m, S2h-i), indicating that MI-503 may affect G3BP1 expression by inhibiting KMT2D expression. The half-life of AR was significantly shortened after MI-503 treatment (Fig. 3k, l, n, and o), which further confirmed a reduction in AR protein stability. These results suggest that MI-503 may inhibit the activity of the AR signaling pathway by accelerating AR degradation.

### Combination of MI-503 and enzalutamide synergistically inhibits PCa

**Fig. 4:**
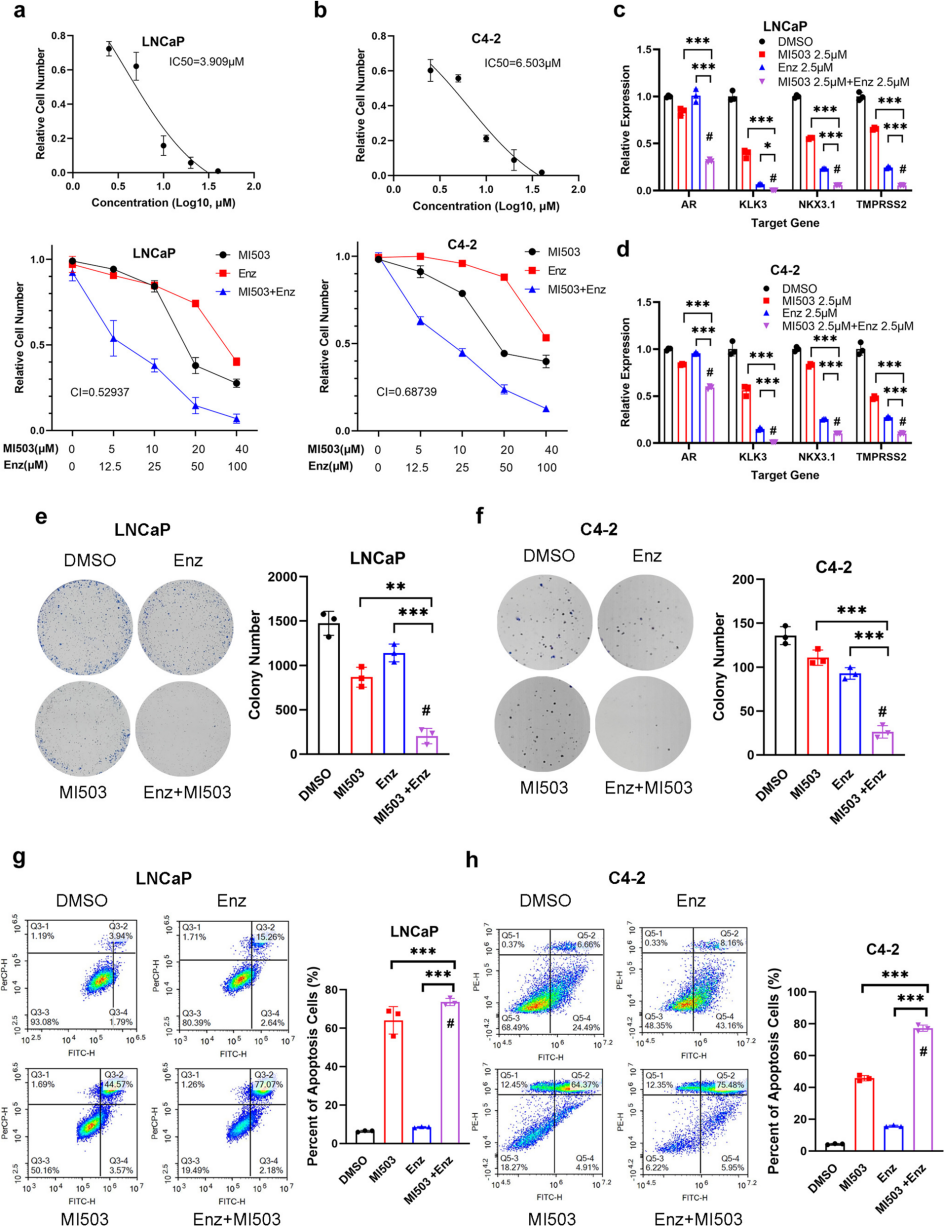
Combination of methylation inhibitor MI-503 and enzalutamide synergistically inhibits prostate cancer in vitro. **a**, **b** IC_50_ of MI-503 in LNCaP (**a**) and C4-2 (**b**) cells and the combination index with enzalutamide. **c**, **d** Effects of MI-503 combined with enzalutamide on the androgen receptor (AR) and the mRNA levels of its downstream genes in LNCaP (**c**) and C4-2 (**d**) prostate cancer cells. *GAPDH* was used as the internal reference gene. **e**–**f** Colony formation assay for assessing the inhibitory effect of MI-503 combined with enzalutamide on the proliferation of LNCaP (**E**) and C4-2 (**f**) cells. **g**, **h** Flow cytometry analysis of the effect of MI-503 combined with enzalutamide on apoptosis in LNCaP (**g**) and C4-2 (**h**) cells. *P < 0.05; **P < 0.01; ***P < 0.001. # was considered synergistic by Bliss-independent analysis. All experiments were conducted in triplicate, and representative data are presented. Enz: Enzalutamide

Enzalutamide is an anti-androgen therapeutic drug widely used in clinical practice. It specifically inhibits the binding of androgens to AR, prevents AR from entering the nucleus, and inhibits the transcription of genes downstream of AR. Therefore, we investigated the synergistic effect of MI-503 and enzalutamide on PCa. We first examined the half-maximal inhibitory concentration (IC_50_) of MI-503 in the two PCa cell lines. In LNCaP and C4-2 cells, the IC_50_ values ​​of MI-503 were 3.909 and 6.503 μM, respectively (Fig. 4a, b). The synergistic effect of the drug combination was evaluated by calculating the combination index (CI). In both the cell lines, the CI values ​​ for the MI-503 and enzalutamide combination were < 1, indicating a synergistic effect in LNCaP and C4-2 cells (Fig. 4a, b). We further explored the combined effect of MI-503 and enzalutamide in synergistically inhibiting the transcription of the *AR* and its downstream genes. Treatment with enzalutamide alone markedly suppressed the transcription of AR downstream genes relative to the control group, aligning with its established pharmacological activity. The inhibitory effect of the MI-503 and enzalutamide combination on the transcription levels of these genes was further enhanced synergistically (Fig. 4c, d). In 22Rv1 cells, the combination also promoted the degradation of AR-V7 (Fig. S1h). The colony formation assay demonstrated that combined treatment markedly suppressed tumor cell proliferation, leading to fewer and smaller colonies compared with the control group. In contrast, although the experimental groups treated with enzalutamide or MI-503 alone showed a certain degree of inhibitory effect, the effect did not reach the level achieved for the combined drug group (Fig. 4e, f). Apoptosis is a key indicator of drug-induced tumor suppression. Combined treatment led to enhanced apoptosis in PCa cells relative to single-agent treatments or the drug-free control group (Fig. 4g, h).

**Fig. 5:**
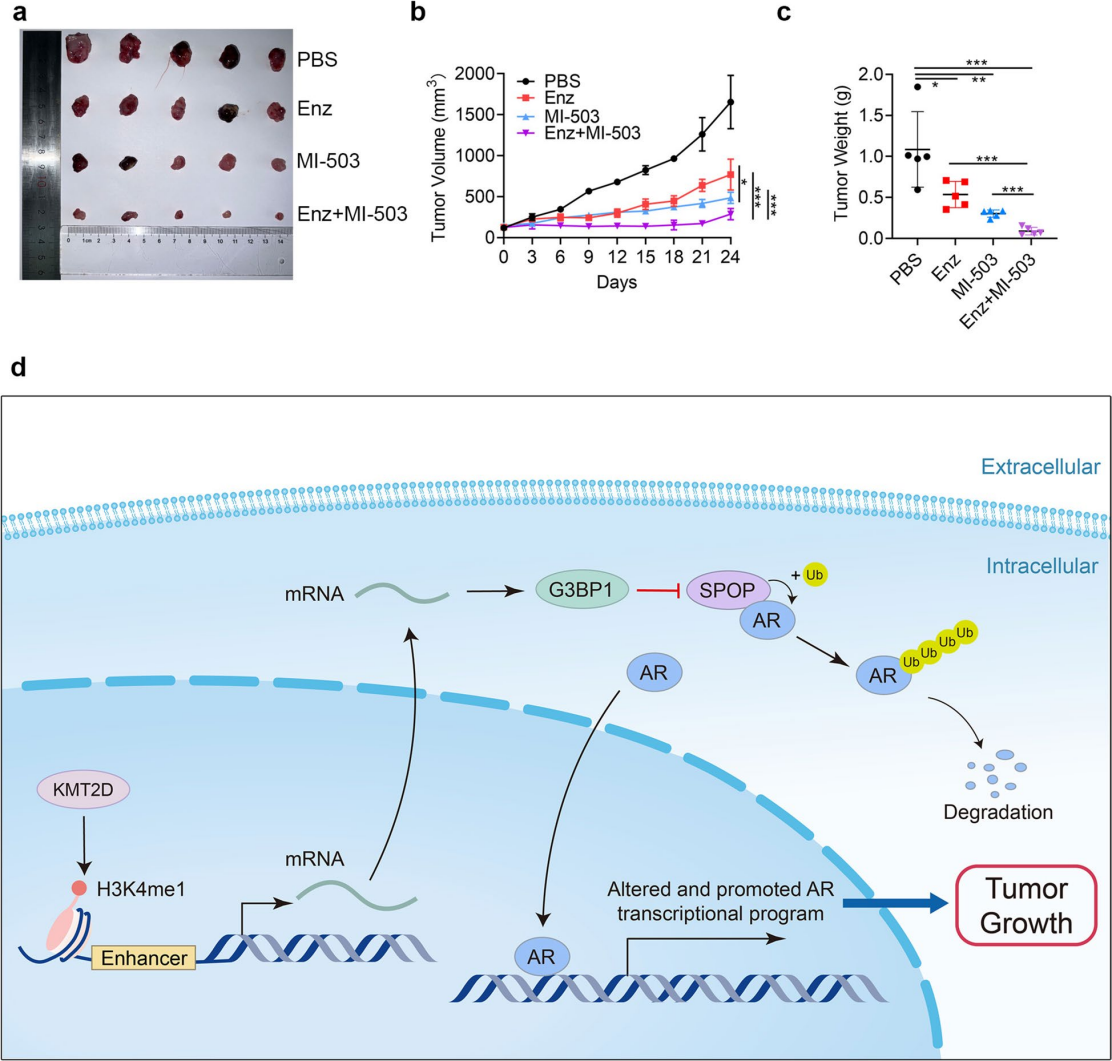
The MI-503 and enzalutamide combination synergistically inhibits prostate cancer in vivo. **a**–**c** Tumor images (**a**), tumor growth curves (**b**), and tumor weights (**c**) of C4-2 xenografts from mice treated with phosphate-buffered saline (PBS), enzalutamide, MI-503, or both. **d** Schematic representation of the mechanism by which KMT2D promotes SPOP inhibition via G3BP1, leading to the activation of androgen receptor (AR) and prostate cancer progression. *P < 0.05; **P < 0.01; ***P < 0.001. All experiments were conducted in triplicates, and representative data are presented. Enz: Enzalutamide

Based on previous cell function experiments, we conducted an in vivo tumor formation experiment in nude mice to further verify the antitumor effects of the MI-503 and enzalutamide combination. Compared with enzalutamide monotherapy, combined treatment with MI-503 and enzalutamide markedly suppressed tumor growth, indicating enhanced antitumor efficacy (Fig. 5a, c). Based on tumor growth curves generated from the recorded measurements, statistical analysis further confirmed that the combination group exhibited a significantly slower tumor growth rate than the single treatment group, supporting the enhanced antitumor efficacy of MI-503 combined with enzalutamide (Fig. 5b). This synergistic effect manifested as statistically significant tumor growth inhibition (*P* < 0.05), while no significant body weight loss was observed during treatment, suggesting the absence of obvious drug toxicity (Fig. S3). In summary, these results indicate that application of the methylation inhibitor MI-503 in CRPC promotes the ubiquitination and degradation of AR and increases the therapeutic effect of AR inhibitors.

## Discussion

In this study, we unveiled a novel regulatory mechanism for AR degradation wherein KMT2D indirectly regulates the ubiquitination and degradation of AR. We identified a unique KMT2D–G3BP1–SPOP–AR ubiquitin-signaling axis that amplifies AR signaling. Notably, KMT2D is one of the most frequently mutated proteins in patients with PCa, and its high expression is associated with a poor prognosis [15]. Thus, KMT2D emerges as a potential intervention target for PCa. We also explored a novel combination therapy of the histone methyltransferase inhibitor MI-503 and enzalutamide in AR-positive and AR splice variant-positive cell lines. Our results confirmed the synergistic therapeutic effects of this combination, which can continue to inhibit the AR signaling pathway during the CRPC stage, thereby delaying disease progression and improving the prognosis of patients with PCa.

KMT2D is a multifunctional protein that acts as a proto-oncogene in PCa. We discovered that KMT2D indirectly regulates the post-translational modifications of AR besides regulating the activity of AR as a transcription factor and histone methyltransferase. Notably, AR stability is primarily regulated by ubiquitination and deubiquitination processes. Based on current understanding, Ring finger protein 6(RNF6), Siah E3 ubiquitin protein ligase 2(SIAH2), SPOP, MDM2, SKP2, and STUB1 (CHIP) are the main E3 ubiquitin ligases that mediate the regulation of AR ubiquitination [21–24]. RNF6 and Siah2 ubiquitinate AR and further increase their transcriptional activity, whereas the other four mainly cause ubiquitinated AR to bind to the proteasome and undergo degradation [25–28]. Our results indicate that KMT2D-mediated regulation of AR stability is associated with its post-translational regulation. In KMT2D-knockdown cells, the half-life of AR was significantly shortened, indicating that KMT2D regulates protein levels by inhibiting AR degradation. Moreover, application of the proteasome inhibitor MG132 partially restored the AR protein levels, further confirming the regulatory effect of KMT2D on ubiquitination and degradation of AR. No changes in the expression of the above-mentioned E3 ubiquitin ligases were detected after KMT2D knockdown; however, the binding of SPOP to AR increased significantly, which is indicative of the existence of an intermediary between KMT2D and SPOP, and suggests that KMT2D regulates AR stability by modulating the degradation activity of SPOP.

G3BP1 is believed to interact with the SPOP–CUL3 complex, and this interaction may inhibit the binding of SPOP to AR, thereby reducing the ubiquitination levels of AR [19, 29]. Our data further indicated that G3BP1 is the downstream target of KMT2D. Analysis of Cancer Genome Atlas (TCGA) PCa data demonstrated that *G3BP1* expression was positively correlated with *KMT2D* expression. Moreover, the CUT&Tag showed the binding of KMT2D to the enhancer region of G3BP1, whereas ATAC-seq and PCR analysis indicated that this binding mediated the transcriptional regulation of G3BP1. These results indicated that KMT2D epigenetically activates G3BP1, which in turn inhibits the ubiquitination and degradation of AR, promoting its overexpression in CRPC.

In the CRPC stage, AR continues to play a dominant role in driving disease progression, making the inhibition of reactivated AR a crucial therapeutic strategy for PCa at this stage [30, 31]. In previous studies, we found that combining second-generation AR antagonists with AR degraders could achieve synergistic therapeutic effects by antagonizing the AR transcriptional activity and expression levels, and disrupting the interaction between AR and AR splice variants, thereby effectively blocking the reactivated AR and inhibiting disease progression [32, 33]. In our in vitro experiments, we found that MI-503 affected G3BP1 expression and accelerated AR degradation by inhibiting KMT2D, which supported our contention regarding the potential synergistic effects of the MI-503 and enzalutamide combination. Consistent with this, in vivo tumor growth was significantly inhibited in mice treated with this combination, indicating that the combined use of these drugs may offer enhanced antitumor activity and provide new strategies for clinical treatment.

Our study had several limitations. First, similar to *KMT2D*, *SPOP* is frequently mutated in PCa with a mutation frequency of approximately 10% [34]. After *SPOP* mutation, the regulatory relationship between SPOP and G3BP1 may change, which could result in the regulation of G3BP1 by KMT2D no longer affecting AR expression. Therefore, in the future, clinical cases need to be analyzed to further investigate the KMT2D–G3BP1–SPOP–AR axis under mutated conditions. Second, regarding the combination therapy, we only explored enzalutamide and tested it in LNCaP, C4-2, and 22Rv1 cell lines. Future work should expand the scope of drugs to include other novel AR antagonists, such as apalutamide and darolutamide, and incorporate enzalutamide-resistant strains, and patient-derived sample or xenograft models, for a more comprehensive evaluation of the therapeutic strategy.

In summary, our study uncovers a novel role of KMT2D in PCa, demonstrating that it epigenetically regulates the SPOP–G3BP1 axis to mediate AR reactivation in CRPC. These findings not only enhance our understanding of PCa progression but also introduce a promising therapeutic strategy combining MI-503 and enzalutamide, providing new treatment avenues for patients with advanced PCa.

## Materials and methods

### Cell culture and transfection

C4-2, LNCaP, and 22Rv1 cell lines were purchased from Procell Life Science and Technology Co., Ltd. (Guangzhou, China). All cell lines were cultured in RPMI-1640 medium (Procell; Cat. No. PM150110) supplemented with 10% fetal bovine serum (Procell; Cat. No. 164210) and 1% penicillin/streptomycin solution (Procell; Cat. No. 15140122). Cells were cultured at 37 °C in a humidified incubator with 5% CO_2_. Short tandem repeat analysis was used to confirm cell line identity, and mycoplasma tests confirmed that the cultures were contamination-free.

Enzalutamide (MedChemExpress; Cat. No. SC0074), MI-503 (AbMole; Cat. No. MI-503), cycloheximide (MedChemExpress; Cat. No. HY-12320), and MG132 (MedChemExpress; Cat. No. HY-13259) were dissolved in dimethyl sulfoxide (DMSO; Beyotime; Cat. No. ST038) to prepare stock solutions.

For gene silencing experiments, C4-2 and LNCaP cells were seeded in 6-well plates at a density of 2 × 10^5^ cells per well approximately 24 h prior to transfection. Transfection was performed using non-targeting control siRNA (siNC), KMT2D siRNA, or SPOP siRNA. siRNA (50 nM) was mixed with the Lipofectamine™ 3000 transfection reagent (Invitrogen; Cat. No. L3000015) in Opti-MEM™ I Reduced Serum Medium (GIBCO; Cat. No. 31985–070) at recommended ratios (e.g., 1.5 µL reagent per well for a 6-well plate) following the manufacturer’s instructions. The transfection complexes were prepared and kept at room temperature for 15 min before gentle addition to the cells. The cells were then incubated for 48–72 h and collected for subsequent analysis.

### Cell viability assay

The Cell Counting Kit-8 (CCK-8; Dojindo; Cat. No. CK04) was employed to measure cell growth in accordance with the manufacturer’s protocol. LNCaP and C4-2 cells were seeded in 96-well microplates at 1,000 cells per well. Cells were incubated for 12 h to permit attachment, after which the medium was changed to include various concentrations of MI-503 and/or enzalutamide. The cells were further cultured for 24, 48, or 72 h, as indicated in the results, to ensure that the absorbance readings were within the linear range. Subsequently, CCK-8 reagent was added to the plates and incubated according to the manufacturer’s guidelines, and a microplate reader (Tecan Trading AG) was used to measure the optical density (OD) at 450 nm.

### Colony formation assay

LNCaP and C4-2 cells were seeded in 6-well plates at 1,000 cells per well. Cells were maintained in medium supplemented with varying concentrations of MI-503 and/or enzalutamide. The medium containing the corresponding drugs was replaced every 3–4 days. After a 14-day incubation period, the cells were washed twice with phosphate-buffered saline (PBS, Procell; Cat. No. PB180523), fixed with 4% paraformaldehyde (Beyotime; Cat. No. P0099) for 15 min at room temperature, and then stained with 0.1% crystal violet solution (Beyotime; Cat. No. C0121) for 20 min at room temperature. The cells were then thoroughly washed with tap water until the background was clear. Visible colonies were subjected to image analysis and quantification using the ImageJ software.

### Immunoprecipitation

After reaching 80–90% confluency in 10 cm dishes, the cells were washed twice with ice-cold PBS and then lysed with RIPA buffer (Beyotime; Cat. No. P0013B) containing protease inhibitor cocktail (MedChemExpress; Cat. No. HY-K0010) and phosphatase inhibitors (MedChemExpress; Cat. No. HY-K0021). Following 30 min incubation on ice with occasional agitation, lysates were centrifuged at 12,000 × *g* for 10 min at 4 °C to obtain clarified supernatants. Protein quantification was performed using a BCA protein assay kit (Beyotime; Cat. No. P0010).

Total protein (500–1000 µg) was precleared with Protein A/G Agarose (MedChemExpress; Cat. No. HY-K0202) for 1 h at 4 °C to minimize nonspecific interactions. The precleared lysates were then incubated overnight (12–16 h) with specific primary antibodies with gentle rotation at 4 °C. The next day, fresh Protein A/G Agarose (MedChemExpress; Cat. No. HY-K0202) was added, and incubation was continued with rotation for 6 h at 4 °C. Following incubation, low-speed centrifugation was used to collect the beads, which were then washed three times with ice-cold lysis buffer (5 min, each wash). The bound proteins were eluted by resuspending the beads in SDS loading buffer and heating at 95 °C for 5 min, followed by western blot.

### Western blotting

For each lane, 20–30 µg of total protein was combined with SDS loading buffer (Beyotime; Cat. No. P0015). Proteins were separated via SDS–polyacrylamide gel electrophoresis (SDS-PAGE) on 10% polyacrylamide gels and then transferred onto a nitrocellulose membrane (abm; Cat. No. B500) using a wet transfer system (Bio-Rad; Model 1,703,930) at 100 V for 1 h with transfer buffer (Beyotime; Cat. No. P0021A).

To block nonspecific binding, membranes were treated with 5% skim milk (Biosharp; Cat. No. BS102) in PBST (0.1% Tween-20 in PBS) for 1 h at room temperature, followed by overnight incubation at 4 °C with specific primary antibodies. Details of the antibodies and their working dilutions are listed below: AR (Santa Cruz Biotechnology Inc.; Cat. No. sc-7305; 1:1000 dilution), SKP2 (Proteintech; Cat. No. 15010–1-AP; 1:1000 dilution), SPOP (Proteintech; Cat. No. 16750–1-AP; 1:1000 dilution), STUB1 (Proteintech; Cat. No. 16750–1-AP; 1:1000 dilution), MDM2 (Cell Signaling Technology; Cat. No. 86934S; 1:1000 dilution), Ubiquitin (ZEN BIO; Cat. No. 382766; 1:1000 dilution), and Glyceraldehyde-3-phosphate dehydrogenase (GAPDH) (Santa Cruz Biotechnology Inc.; Cat. No. sc-47724; 1:1000 dilution). GAPDH was used as an internal loading control for protein normalization.

The following day, after being washed with PBST, the membranes were incubated with corresponding HRP-conjugated secondary antibodies (ABclonal; Cat. No. AS014; dilution 1:20,000) for 1 h at room temperature. After an additional three washes with PBST, immunoreactive bands were detected using an enhanced chemiluminescence system (Biosharp; Cat. No. BL523B) and visualized with a chemiluminescence imaging system (Tanon; Model 5200). Image analysis and densitometric quantification were performed using the ImageJ software.

### Determination of half-life of protein

To determine protein half-life, LNCaP and C4-2 cells were seeded at a density of 2 × 10^5^ cells per well in 6-well plates. After 24 h, 100 µg/mL cycloheximide (CHX) was added to the cell culture medium to inhibit protein synthesis, and total proteins were extracted and analyzed by western blotting to assess the degradation rate of the target proteins. Protein levels were quantified using the ImageJ software, and degradation curves were plotted to calculate the protein half-life.

### Quantitative real-time PCR

Total RNA was isolated from cells using the TRIzol reagent (Invitrogen; Cat. No. 15596026) according to the manufacturer’s instructions. One microgram of total RNA was used for complementary DNA (cDNA) synthesis using HiScript® III All-in-one RT SuperMix (Vazyme; Cat. No. R323-01). Each cDNA sample was amplified using Taq Pro Universal SYBR quantitative PCR Master Mix (Vazyme Bio; Cat. No. Q712-02) on a QuantStudio 5 real-time PCR system (Thermo Fisher Scientific Co., Ltd). Gene expression was quantified using the comparative 2^−△△Ct^ method [35]. *GAPDH* was used as an internal reference gene for normalization. Data analysis was performed using the QuantStudio™ Design & Analysis Software (v1.5.1). The sequences of primers used in the study are provided in the supplementary materials.

### Cellular thermal shift assay (CETSA)

CETSA experiments were performed on live LNCaP, C4-2, and 22Rv1 cells following a previously described universal CETSA protocol. Cells were washed twice with PBS and then resuspended in serum-free medium. The cells were then divided into different groups and treated with vehicle control (DMSO) or varying concentrations of test compounds for 60 min at 37 °C. Thereafter, cell suspensions were aliquoted into PCR tubes and subjected to thermal stress at different temperatures for 3 min. Following thermal stress, the samples were cooled for 3 min at ambient temperature. The protease inhibitor (MedChemExpress; Cat. No. HY-K0010) was added before cell lysis, which was performed by repeated cycles of freezing in liquid nitrogen and thawing in heating block. The lysates were subsequently centrifuged at 20,000 × *g* for 20 min at 4 °C to remove cellular debris. The resulting supernatant was carefully transferred to new tubes and immediately subjected to western blot analysis.

### Flow cytometry for apoptosis detection

Apoptosis was detected using the Annexin V- Fluorescein isothiocyanate (FITC) Apoptosis Detection Kit (Beyotime; Cat. No. C1062) and propidium iodide (PI) staining according to the manufacturer’s instructions. Treated cells were collected via trypsin digestion (Beyotime; Cat. No. C0203) and washed twice with ice-cold PBS (200 × *g*, 5 min). Cell pellets were resuspended in 1X binding buffer (provided in the kit), adjusting the cell density to 1 × 10^⁶^ cells/mL. Annexin V-FITC and PI were added, and the mixture was kept in the dark at room temperature for 15 min. Subsequently, binding buffer was added to each tube, and cells were analyzed via flow cytometry within 1 h. Data analysis was performed using the FlowJo software, and the percentage of apoptotic cells was calculated using standard gating strategies.

### CUT&Tag and data analysis

LNCaP and C4-2 cells were transfected with siNC or siKMT2D for 48 h. For CUT&Tag experiments, two independent biological samples, each containing 5 × 10^5^ live cells, were prepared following the guidelines provided with the CUT&Tag Assay Kit (Vazyme; Cat. No. TD903-01). The cells were washed with PBS and then incubated in cell lysis buffer (20 mM HEPES (pH 7.9), 10 mM KCl, 0.1% Triton X-100, 20% glycerol) on ice for 10 min to isolate nuclei. Subsequently, nuclei were bound to ConA Beads and incubated with rotation for 10 min at 4 °C. After washing the beads with wash buffer, antibody buffer containing 0.5 µg of specific antibody was added, and samples were incubated overnight at 4 °C. The next day, beads were washed with wash buffer, 2.5 µL of pAG-NMase was added, and the mixture was incubated for 10 min at room temperature. After further washing, 1 µL of chromatin digestion enhancer was added, and samples were stored at 4 °C for 2 h. The reaction was terminated by adding stop buffer and 0.5 ng of Spike-in DNA and incubating for 10 min at 37 °C. DNA was isolated using a DNA purification kit (EpiCypher; Cat. No. 14–1001). For library construction, 5 ng of purified DNA was processed using an Illumina Library Prep Kit (Vazyme; Cat. No. TD502-01). The libraries were purified using 1.0X AMPure beads, and the size distribution of library fragments was determined using Agilent Bioanalyzer. The libraries were sequenced by Novogene (Beijing, China) to a depth of 20 million reads per library, with 150 bp paired-end reads. The quality of sequencing reads was assessed using FastQC. Adapter sequences were trimmed using Trim Galore. Reads with quality scores > 30 were mapped to the human reference genome hg38 using Bowtie2. Sequencing data were scaled using normalization factors derived from *E. coli* Spike-in DNA read sequencing depth. After mapping to the hg38 genome, alignment results were converted from SAM to BAM format, and PCR duplicates were removed.

### Preparation of ATAC-Seq library and data analysis

Nuclei were isolated and purified from sample material. The resulting nuclear pellet was subsequently resuspended in Tn5 transposase reaction mix and reacted for 30 min at 37 °C. After transposition, equimolar amounts of Adapter1 and Adapter2 were introduced, and PCR was performed to amplify the DNA libraries. The libraries were purified using AMPure beads (Beckman; Cat. No. A63881), and library integrity was assessed using a Qubit fluorometer (Thermo Fisher Scientific; Model Q32884). Indexed samples were clustered on a cBot Cluster Generation System using a TruSeq PE Cluster Kit v3-cBot-HS (Illumina), following the manufacturer’s guidelines. After cluster formation, libraries were sequenced on an Illumina sequencing platform at Novogene (Beijing, China), generating 150 bp paired-end reads. Raw sequencing data were processed using fastp (version 0.20.0) to filter clean reads and remove adapters, poly N sequences, and low-quality sequences. Quality metrics, such as Q20, Q30, and GC content, were also determined. The human reference genome (hg38) and annotation data were used, and BWA (version 0.7.12) was employed to build an index for aligning clean reads. Mitochondrial and chloroplast DNA reads, incorrectly paired reads, and PCR duplicates were excluded from the analysis.

## Mouse model

Four-week-old male BALB/c nude mice were purchased from GemPharmatech Co. Ltd. (Nanjing, China). Under specific pathogen-free conditions, mice were maintained in a controlled environment with a 22 ± 2 °C temperature, 50–70% humidity, and a 12-h light/dark cycle. They were supplied with food and water ad libitum, and their body weight and overall health were assessed daily. Animal handling was performed according to protocols approved by the Animal Care and Use Committee of Ganzhou People's Hospital.

For C4-2 subcutaneous tumor xenografts, 2 × 10^6^ C4-2 cells mixed with Matrigel (1:1 ratio, YEASEN; Cat. No. 40183ES08) were subcutaneously inoculated into the flanks of nude mice using a 27G syringe (*n* = 5 per group). After tumor cell inoculation, experimental mice underwent orchiectomy to simulate castrate conditions. Subsequently, mice were randomly divided into four groups: control (PBS), enzalutamide alone (10 mg/kg/day), MI-503 alone (5 mg/kg/day), and combination treatment with MI-503 (5 mg/kg/day) and enzalutamide (10 mg/kg/day). Enzalutamide was administered via oral gavage and MI-503 was administered via intraperitoneal injection. Drug treatment commenced when tumor volumes reached approximately 100 mm^3^. Tumor dimensions were recorded at two-day intervals using calipers, and the volume of each tumor was computed from the longest (L) and shortest (W) diameters based on the following equation: Volume = (L × W^2^)/2. Treatment continued until tumor volume reached a predetermined size of 2000 mm^3^ or when mice showed signs of severe illness, at which point mice were humanely euthanized. Animals were euthanized via CO_2_ inhalation, and death was confirmed by checking for 2 min of respiratory arrest and lack of reflexes. Tumors were excised, their weight and volume were measured, and photographs were taken to assess the treatment efficacy. Tumor data from each group of mice were collected for statistical analysis, and tumor growth curves were plotted.

### Correlation analysis using cBioPortal

Correlation analysis between *KMT2D* and *G3BP1* mRNA expression was performed using data from TCGA Prostate Adenocarcinoma cohort via the cBioPortal for Cancer Genomics platform (www.cbioportal.org) on Aug 2023. Expression values were retrieved as log2-transformed Transcripts Per Million. Pearson correlation coefficients (*R*) and associated *P*-values were automatically calculated and displayed using the cBioPortal platform.

### Statistical analysis

All data are presented as mean ± standard deviation. Statistical analysis was performed using SPSS 26.0 (IBM Corp.). Differences between individual groups were analyzed using one-way analysis of variance (ANOVA). Statistical significance was set at *P* < 0.05. Interactions between the two drugs were determined by calculating CI using the Compusyn software. Synergistic effects were further evaluated using two-way ANOVA and the Bliss independence model. For all statistical analyses, differences were marked as ns (not significant); *P < 0.05; **P < 0.01; ***P < 0.001. # was considered synergistic by Bliss independence analysis. All experiments were performed with at least three biological replicates.

## Supplementary Information

Supplementary Material 1.

## Data Availability

Data, scripts, and codes are available upon request. The data that support the findings of this study are available from the corresponding author upon reasonable request.
