# Supplementary material for: Histone methyltransferase KMT2D targets the SPOP-G3BP1 axis to enhance AR stability and drive castration-resistant prostate cancer progression
Source: Mol Biomed. 2025 Nov 17;6:112. doi: 10.1186/s43556-025-00354-8 (PMC12623541; doi:10.1186/s43556-025-00354-8)
Supplement: Supplementary file 1 — Supplementary Material 1. [file 43556_2025_354_MOESM1_ESM.docx]

**Supplemental information**

**Histone methyltransferase KMT2D targets the SPOP-G3BP1 axis to enhance AR stability and drive castration-resistant prostate cancer progression**

Haoran Wen^1,3^, Maierhaba Maheremu^1^, Kaidi Zhang^1,3^, Liuru Bao^1^, Mayao Luo^1,3,4^, Yifan Zhang^1,4^, Yuanpeng Liao^1,3^, Manli Zhou^1,3^, Chenwei Wu^1^, Shidong Lv^1,4*,^ Xiaofu Qiu^2*^ and Qiang Wei^1,3,4*^

^1^Department of Urology, Nanfang Hospital, Southern Medical University, Guangzhou, Guangdong 510515, China.

^2^The Affiliated Guangdong Second Provincial General Hospital of Jinan University, Guangzhou, 510317, China.

^3^Department of Urology, Ganzhou Hospital-Nanfang Hospital, Southern Medical University, Ganzhou, 341000, China.

^4^Department of Urology, Guangdong Cardiovascular Institute, Guangdong Provincial People's Hospital, Guangdong Academy of Medical Sciences, Southern Medical University, Guangzhou, Guangdong, 510080, China.

*Correspondence

Qiang Wei, Department of Urology, Nanfang Hospital, Southern Medical University, Guangzhou, Guangdong, 510515, China.

E-mail: qwei@smu.edu.cn

Phone: +8620-61641765

Xiaofu Qiu, The Affiliated Guangdong Second Provincial General Hospital of Jinan University, Guangzhou, 510317, China.

The Second School of Clinical Medicine, Southern Medical University, Guangzhou, 510515, China

E-mail: xfqiu123@163.com

Phone: +86138 8990 5577

Shidong Lv, Department of Urology, Nanfang Hospital, Southern Medical University, Guangzhou, Guangdong, 510515, China.

E-mail: lsd990@smu.edu.cn

Phone: +8620-61641765

**Fig. S1 KMT2D is upregulated in castration-resistant prostate cancer (CRPC) and regulates AR/AR-V7 expression**

**
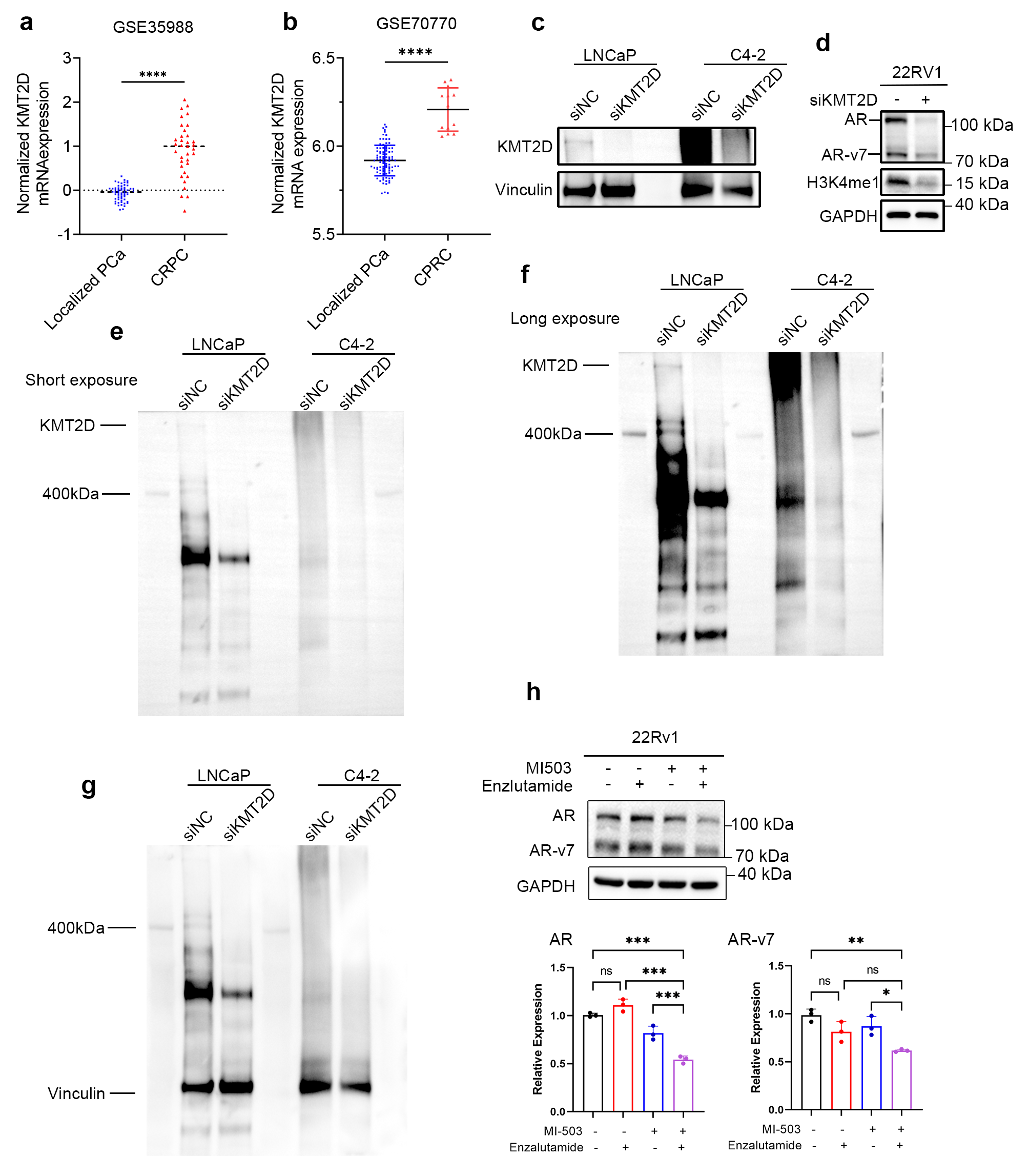
**

**Fig. S1 KMT2D is upregulated in castration-resistant prostate cancer (CRPC) and regulates AR/AR-V7 expression**

(a–b) Normalized *KMT2D* mRNA expression levels in localized prostate cancer (PCa) and CRPC samples from public datasets GSE35988 (a) and GSE70770 (b). Data are shown as mean ± SEM; *****P* < 0.0001 by unpaired *t*-test. (c) Western blot of KMT2D expression in LNCaP and C4-2 cells transfected with siKMT2D or non-targeting siNC. Vinculin was used as a loading control. (d) Western blot of AR, AR-V7, and H3K4me1 in 22Rv1 cells after KMT2D knockdown. GAPDH was used as a marker and loading control. (e–f) Full blot images corresponding to panel (c). (e) Results after short exposure; (f) Results after longer exposure.(g) The same membrane as in (e–f) was reprobed for vinculin as a loading control. (h) Western blot and quantification of AR and AR-V7 expression in 22Rv1 cells treated with MI-503 and/or enzalutamide. GAPDH was used as a loading control. Quantification of protein levels was normalized to GAPDH. **P* < 0.05; ***P* < 0.01; ****P* < 0.001; n.s not significant.

**Fig. S2 Quantification of western blot results corresponding to main text figures**


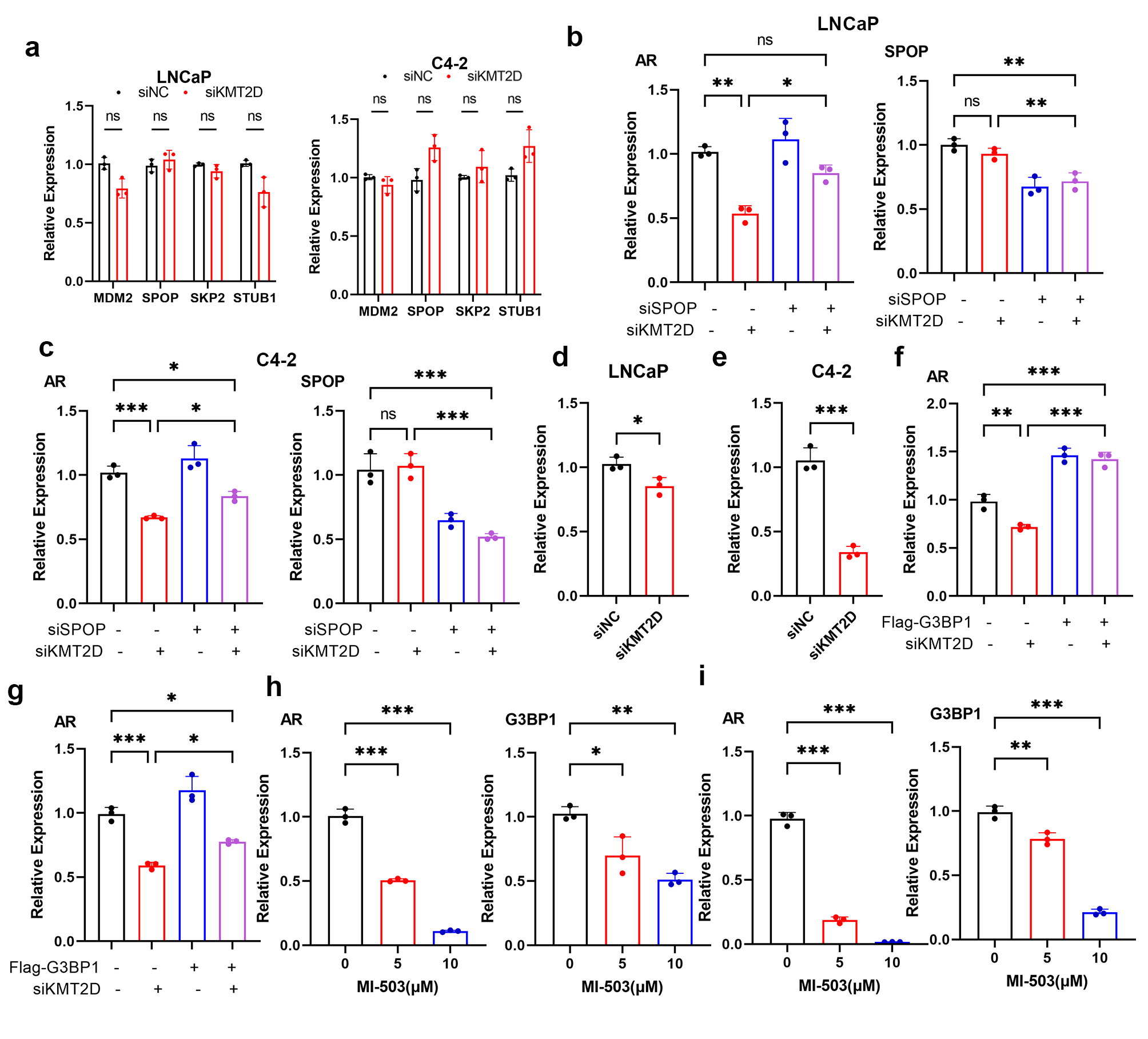


**Fig. S2 Quantification of western blot results corresponding to main text figures**

(a) Quantification of KMT2D protein levels shown in Fig. 2e. (b–c) Quantification of protein expression in Fig. 2g. (b) Results of densitometric analysis for LNCaP cells; (c) Analysis results for C4-2 cells. (d–e) Quantification of western blot bands from Fig. 3b and Fig. 3c, respectively. f–g) Quantitative analysis of protein levels shown in Fig. 3f and Fig. 3g. (h–i) Densitometric quantification corresponding to Fig. 3j–m. All quantifications are based on band intensity normalized to loading controls and presented as mean ± SEM. Statistical significance was calculated using appropriate tests as indicated in the main figure legends; **P* < 0.05; ***P* < 0.01; ****P* < 0.001; ns: not significant

**Fig. S3**


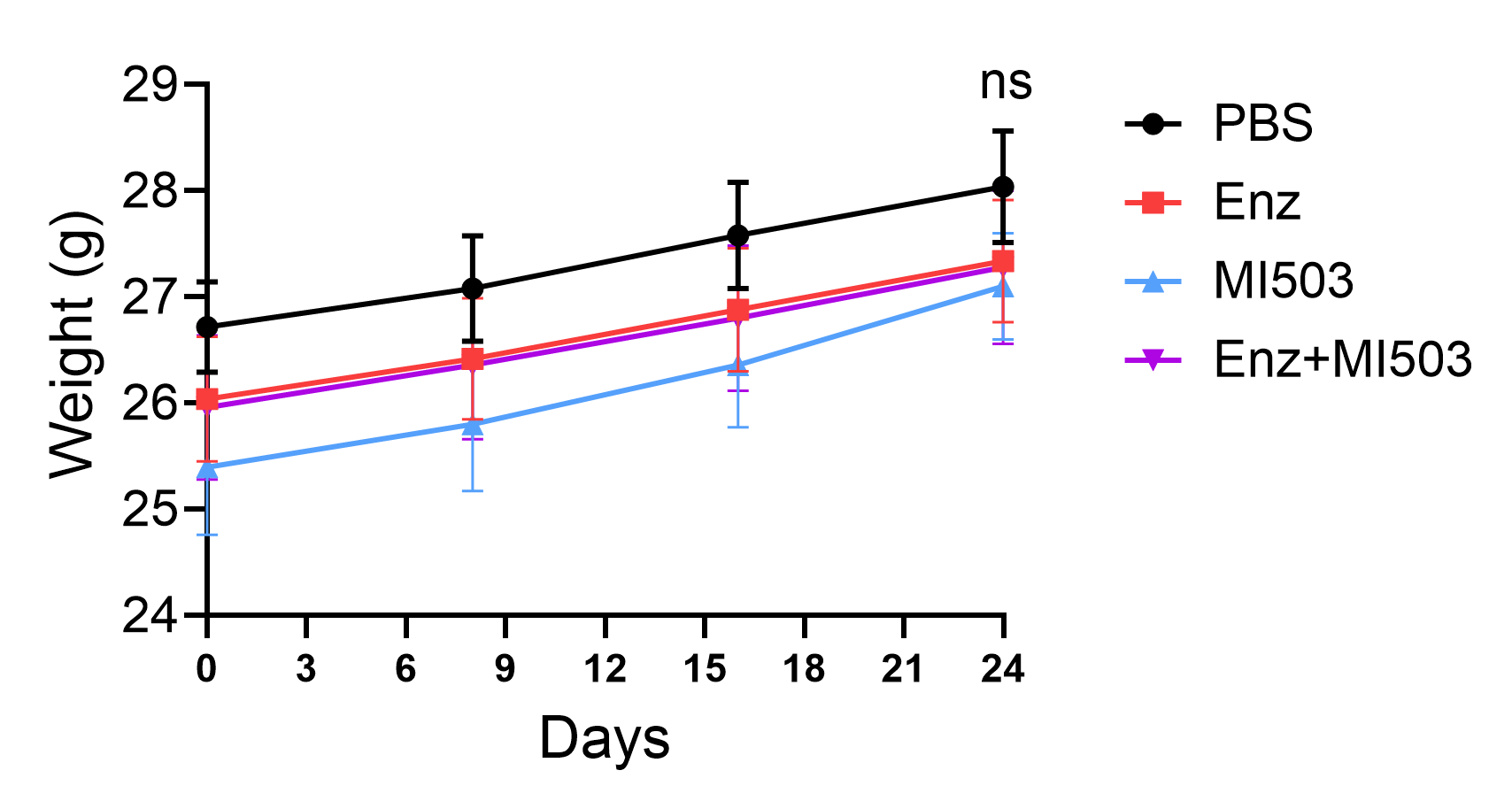


**Fig. S3 Fig. S3 Body weight of mice during treatment**

Mean body weights (g) ± SEM of mice in four treatment groups (PBS, Enz = enzalutamide, MI-503, Enz + MI-503) were recorded every 8 days over a 24‑day treatment period (days 0, 8, 16, 24). Each group contained n = 5 mice. Data are presented as mean ± SEM. Statistical comparison was performed by two‑way ANOVA with Tukey’s multiple comparisons; ns, not significant. See Methods for dosing schedule and other experimental details.

| **Table S1. Primer sequence of qPCR** | |
| --- | --- |
| KLK3 F | CAGGTGTAGACCAGAGTGTTTC |
| KLK3 R | CTGTGTCCTCAGAGAAATTGAGT |
| TMPRSS2 F | TGCTCCAACTCTGGGATAGA |
| TMPRSS2 R | GGATGAAGTTTGGTCCGTAGAG |
| NKX3-1 F | TCTGACAGGTGAATTGGATGG |
| NKX3-1 R | GATTGGAGCAGGGTTTGTTATG |
| AR F | TCTTGTCGTCTTCGGAAATGT |
| AR R | AAGCCTCTCCTTCCTCCTGTA |
| GAPDH F | CTCCTCACAGTTGCCATGTA |
| GAPDH R | GTTGAGCACAGGGTACTTTATTG |
| MDM2 F | GAATCATCGGACTCAGGTACATC |
| MDM2 R | TCTGTCTCACTAATTGCTCTCCT |
| SPOP F | ATCAAGGTGAAGTATGGGGATGT |
| SPOP R | TCACCTCGCAGAAGAGGGT |
| SKP2 F | GGAAGGGAGTCCCATGAAA |
| SKP2 R | GCTGAAGAGCAAAGGGAGTG |
| STUB1 F | AGCAGGGCAATCGTCTGTTC |
| STUB1 R | CAAGGCCCGGTTGGTGTAATA |
| G3BP1 F | CGGGCGGGAATTTGTGAGA |
| G3BP1 R | TCTGTCCGTAGACTGCATCTG |

| **Table S2. Sequence of siRNA** | | |
| --- | --- | --- |
| siKMT2D | Sense | GCAAAUCGCUAGCAUCAUU |
|  | Antisense | AAUGAUGCUAGCGAUUUGC |
| siG3BP1 | Sense | GAGCGCUUAAAGGUCAUGUTT |
|  | Antisense | ACAUGACCUUUAAGCGCUCTT |

**Table S3. Determination of synergy.** Degree of freedom (DF), F value, and p-value were determined by two-way ANOVA tests for a synergistic effect in combination of MI-503 and enzalutamide. p <0.05 are highlighted yellow. The Bliss independence model [(Bliss, Ann Appl Biol 1939 (28)] was used to determine treatment synergy, in which the combined percentage of inhibition from two independent treatments was predicted as Eab,P= Ea + Eb – EaEb, where a and b are two analyzed treatments, and observed combined percentage of inhibition from the two treatments (Eab, O) greater than the predicted combined percentage of inhibition from the two independent treatments, that is, Eab,O – Eab,P >0 is considered synergistic (highlighted blue).

| Experiment | Cell line | Figure | Combination | Two-way ANOVA | | Bliss independence analysis | | |  |
| --- | --- | --- | --- | --- | --- | --- | --- | --- | --- |
|  |  |  |  | DF | *p*-value | *Eab,P* | *Eab,O* | *Eab,O*-*Eab,P* |  |
|  |  |  |  |  |  |  |  |  |  |
| RT-qPCR | LNCaP | 2C-AR | MI-503 2.5μM + Enz 2.5μM | 1,11 | <0.001 | 0.1527883 | 0.6843333 | 0.531544997 |  |
| RT-qPCR | LNCaP | 2C-KLK3 | MI-503 2.5μM + Enz 2.5μM | 1,11 | <0.001 | 0.973351 | 0.997505 | 0.024154368 |  |
| RT-qPCR | LNCaP | 2C-NKX3.1 | MI-503 2.5μM + Enz 2.5μM | 1,11 | <0.001 | 0.869954 | 0.942495 | 0.072540444 |  |
| RT-qPCR | LNCaP | 2C-TMPRSS2 | MI-503 2.5μM + Enz 2.5μM | 1,11 | <0.001 | 0.838258 | 0.945248 | 0.106990625 |  |
| RT-qPCR | C4-2 | 2D-AR | MI-503 2.5μM + Enz 2.5μM | 1,11 | <0.001 | 0.2008096 | 0.4089668 | 0.208157205 |  |
| RT-qPCR | C4-2 | 2D-KLK3 | MI-503 2.5μM + Enz 2.5μM | 1,11 | <0.001 | 0.914785 | 0.990877 | 0.076092886 |  |
| RT-qPCR | C4-2 | 2D-NKX3.1 | MI-503 2.5μM + Enz 2.5μM | 1,11 | <0.001 | 0.789213 | 0.89683 | 0.107617042 |  |
| RT-qPCR | C4-2 | 2D-TMPRSS2 | MI-503 2.5μM + Enz 2.5μM | 1,11 | <0.001 | 0.868155 | 0.899331 | 0.031176465 |  |
| Colony Formation | LNCaP | 2E | MI-503 0.5μM + Enz 0.5μM | 1,11 | <0.001 | 0.544031 | 0.861243 | 0.317211836 |  |
| Colony Formation | C4-2 | 2F | MI-503 0.5μM + Enz 0.5μM | 1,11 | <0.001 | 0.441879 | 0.806373 | 0.364493224 |  |
| Apoptosis | LNCaP | 2G | MI-503 2.5μM + Enz 2.5μM | 1,11 | <0.001 | 0.671599 | 0.736567 | 0.0649679 |  |
| Apoptosis | C4-2 | 2H | MI-503 2.5μM + Enz 2.5μM | 1,11 | <0.001 | 0.542638 | 0.7725 | 0.229861751 |  |
